# Condylar form alteration on skeletal class II patients that underwent orthognathic surgery: An overview of systematic reviews

**DOI:** 10.4317/jced.56947

**Published:** 2020-07-01

**Authors:** Inês Francisco, Adriana Guimarães, Margarida Lopes, António Lucas, Francisco Caramelo, Francisco Vale

**Affiliations:** 1 DDS, MSc. Assistant Professor, Institute of Orthodontics, Faculty of Medicine, University of Coimbra, Portugal; 2 DDS, MSc. Orthodontic Postgraduate, Institute of Orthodontics, Faculty of Medicine, University of Coimbra, Portugal; 3 PhD. Professor, Institute of Clinical and Biomedical Research of Coimbra (iCBR), Faculty of Medicine of the University of Coimbra, Portugal; 4 DDS, MSc. PhD. Program Director and Head of Department, Institute of Orthodontics, Faculty of Medicine, University of Coimbra, Portugal

## Abstract

**Background:**

Bilateral sagittal split osteotomy (BSSO) is commonly considered as the surgical technique of election for the treatment of skeletal class II with mandibular hypoplasia. After orthognathic surgery, condylar resorption can occur as a surgical relapse, which may affect the temporomandibular joint. Objective: This study aimed to summarise published systematic review that assess if orthognathic surgery with mandibular advancement performed on skeletal class II patients results in condylar form alteration.

**Material and Methods:**

A literature search was performed using the electronic databases PubMed, Web of Science, Cochrane Library, Embase along with several sources of grey literature. Selection Criteria: Inclusion criteria were systematic reviews published until December 2019, of skeletal class II patients aged more than 18 years old that underwent BSSO with mandibular advancement surgery. Data collection: The electronic search identified 37 publications. Four publications fulfilled the inclusion criteria and were included in this meta-analysis. Qualitative assessment of the selected studies was performed using the Assessment of Multiple Systematic Reviews – AMSTAR 2 checklist.

**Results:**

Four systematic reviews were included in this review. Despite its low incidence all studies reported condylar resorption. However, there were methodological limitations in all assessed articles.

**Conclusions:**

The alteration of the condylar form may be a consequence of BSSO with mandibular advancement surgery. Additional high quality prospective research assisted by 3D-imaging technology is needed to allow more definite conclusions.

** Key words:**Evidence-based orthodontics, TMJ, Class II, mandibular advancement, malocclusion, Angle class II.

## Introduction

Condylar form alteration is a common factor associate with development of skeletal relapse (1). This is defined as an irreversible progressive alteration of shape and volume of the mandibular condyles following a bilateral sagittal split osteotomy (BSSO) after orthognathic surgery. The alteration of the condylar form alteration is rare but it is a well-known clinical situation that usually affects the temporomandibular joint (TMJ) (2).

BSSO is commonly considered as the surgical technique of election for the treatment of skeletal class II with mandibular hypoplasia (3). The mandibular advancement tends to cause an anterior position of the condyle within the mandibular fossa, which in turn forces the entire condyle/disc complex to follow the same movement during one period of time. In addition, this new anterior mandibular pose generally requires new accommodation of the surrounding soft tissue with major implications in muscle fibres. It seems clear, that this type of therapy frequently leads to a number of changes in the stomatognathic system (4).

The natural adaptive capacity of the TMJs when exceeded may originate in condylar remodeling (5). However, despite these various changes, little is known about the true effect of protrusion as a predisposing, initiating or perpetuating agent of temporomandibular disorders (TMD).

-Objective

The aim of this systematic review with meta-analysis is to answer the following clinical question according to the PICO model (P, population; I, intervention; C, comparative intervention; O, outcome).

“Does mandibular advancement with sagittal split osteotomy on skeletal class II patients result in temporomandibular disorders determined by condylar form alteration?”

## Material and Methods

This Systematic Review (SR) was based on the guidelines of the PRISMA Statement for reports SRs and meta-analysis of studies evaluating healthcare interventions. We registered it on International Prospective Register of Systematic Reviews (PROSPERO): (CRD 42017080676).

The authors noticed that systematic reviews on this field were already available. Thus, we preferred to carry out a review of existing published systematic reviews. This methodology is becoming usual on literature because permits summarize the extensive scientific knowledge on widely explored research topics, so we opted to include only systematic reviews to perform a meta-analysis.

Selection criteria

1. Study Design: Studies included were systematic reviews.

2. Population: Skeletal class II patients that underwent orthognathic surgery with mandibular advancement.

3. Search Strategy: A literature search was performed in electronic bibliographic databases (PubMed, Web of Science, Cochrane Library, Embase), along with several sources of grey literature.

The search was conducted in December 2019, using the following keywords:

- PubMed: “Malocclusion, Angle Class II”[Mesh] AND (“Mandibular Advancement”[Mesh] OR “Osteotomy, Sagittal Split Ramus”[Mesh]) AND “Condylar Resorption”[All Fields]

- Cochrane Library and Web of Science: (malocclusion, Angle class II OR mandibular advancement) AND (osteotomy, sagittal split ramus) AND (condylar resorption).

- EMBASE: (mandibular advancement surgery OR sagittal split ramus osteotomy) AND (temporomandibular disorders).

The inclusion criteria were systematic reviews and Meta-analysis; performed on adults aged more than 18 years old who underwent BSSO with mandibular advancement surgery.

We excluded case reports and case series, randomized and non-randomized controlled trials, cohort studies, editorials, opinions and studies not specifying the parameters of interest, and publications not fulfilling the inclusion criteria.

The selected publications were imported to EndNote software (Thomson Reuters Software; http://endnote.com; 2016), and the duplicates were removed.

Selection of Studies 

Two review authors (IF, AG) performed the study selection independently and in duplicated. They were not blinded to the identity of the authors or their reported results. Selection of the eligible studies was based on screening of the titles and abstracts. Two reviewers (AL, ML) analyzed the full texts of those that met the eligibility criteria. Any disagreement was resolved by consulting a third reviewer (FV). Reviewers kept a record of all the decisions on study identification.

Qualitative Assessment of Included Studies 

The qualitative assessment of the selected studies was performed using the Assessment of Multiple Systematic Reviews (AMSTAR 2) (https://amstar.ca/mascripts/Calc_Checklist.php) checklists. AMSTAR checklists contain several questions directed only to systematic reviews under evaluation.

Statistical Analysis

The sample size, number of subjects and quantitative assessment of the condylar form alteration was collected from the articles of each systematic review included in this overview. Based on these values a meta-analysis was conducted to determine the incidence of condylar form alteration after BSSO surgery. The analysis was carried out resorting to the R statistical platform, in particular to the “metafor” package (6). The heterogeneity of the studies was assessed with I2 coefficient and the Q test.

## Results

**Figure 1:**
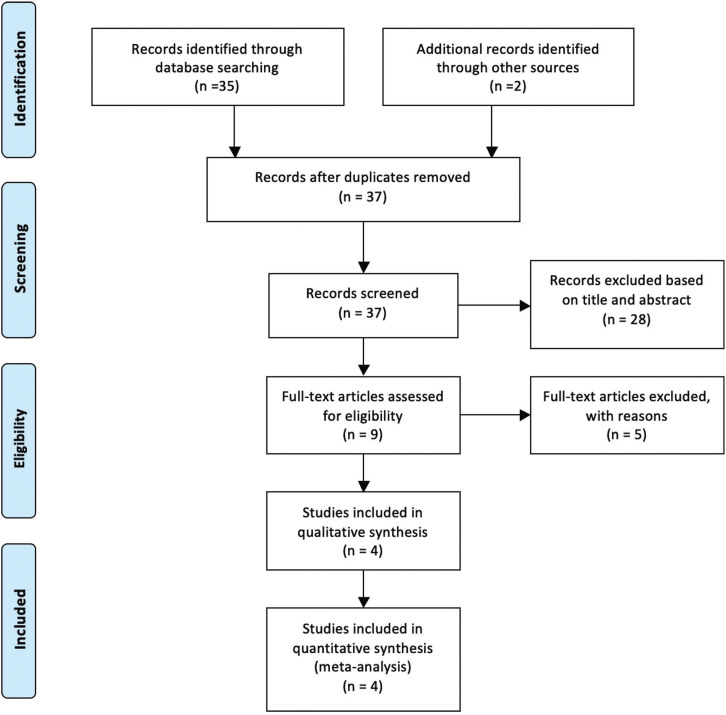
Flow diagram of literature search and screening process.

The electronic searches identified 37 publications. Two review authors examined titles and abstracts of 9 articles were considered potentially relevant (Fig. 1). Full reports were obtained and assessed independently by the review authors. After reading these articles, 5 were excluded by applying the inclusion and exclusion criteria. The main reasons for those items excluded are discriminated in Table 1 (2,7-11).

**Table 1:**
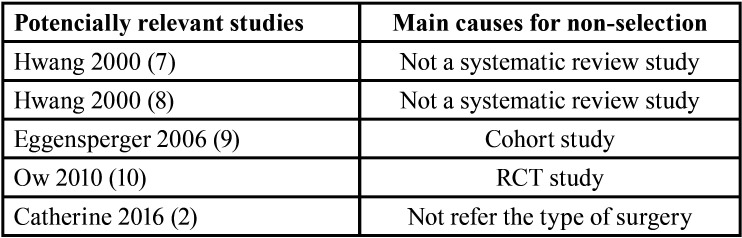
Reasons for studies exclusion.

The 4 selected studies were subjected to qualitative assessment by two review authors (IF, AG). Disagreements were resolved through mediation with a third review (FV). Two studies were considered as low quality of evidence (3,12) and the other two were judged as moderate quality of evidence (4,11), using the AMSTAR 2 checklists.

The detailed results of the accepted publications are explained in  Table 2, Table 3, Table 3 cont. (3,4,11,12). The correlation between condylar remodeling and orthognathic surgery are represented in Table 4 (3,4,11,12).

**Table 2:**
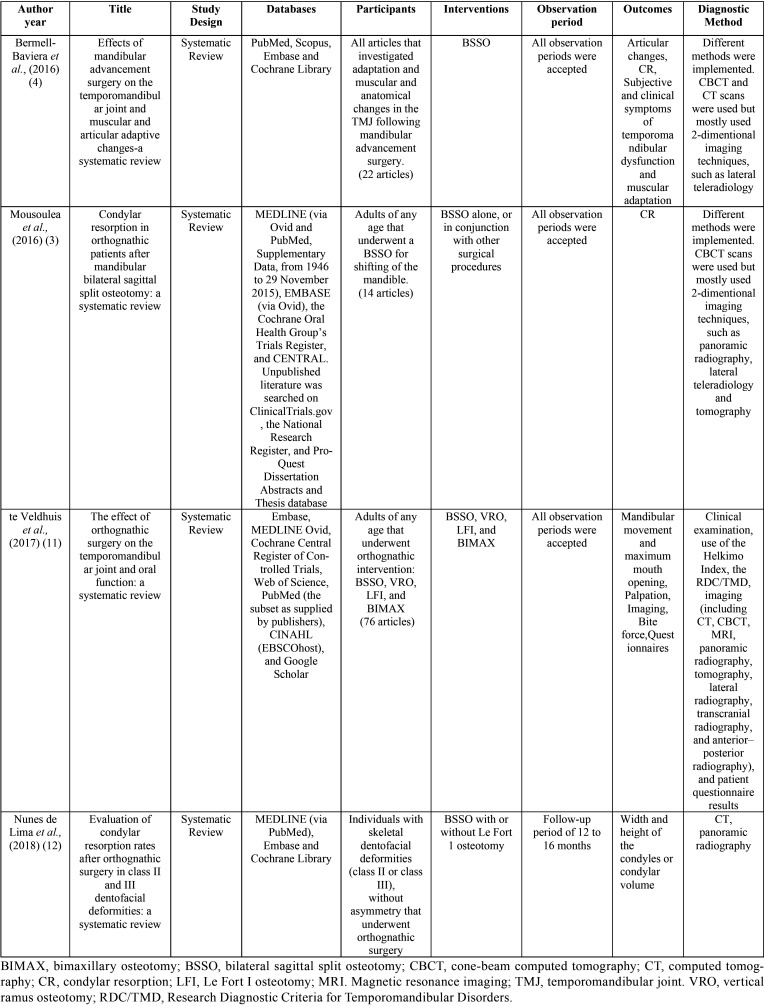
Characteristics of included studies.

**Table 3:**
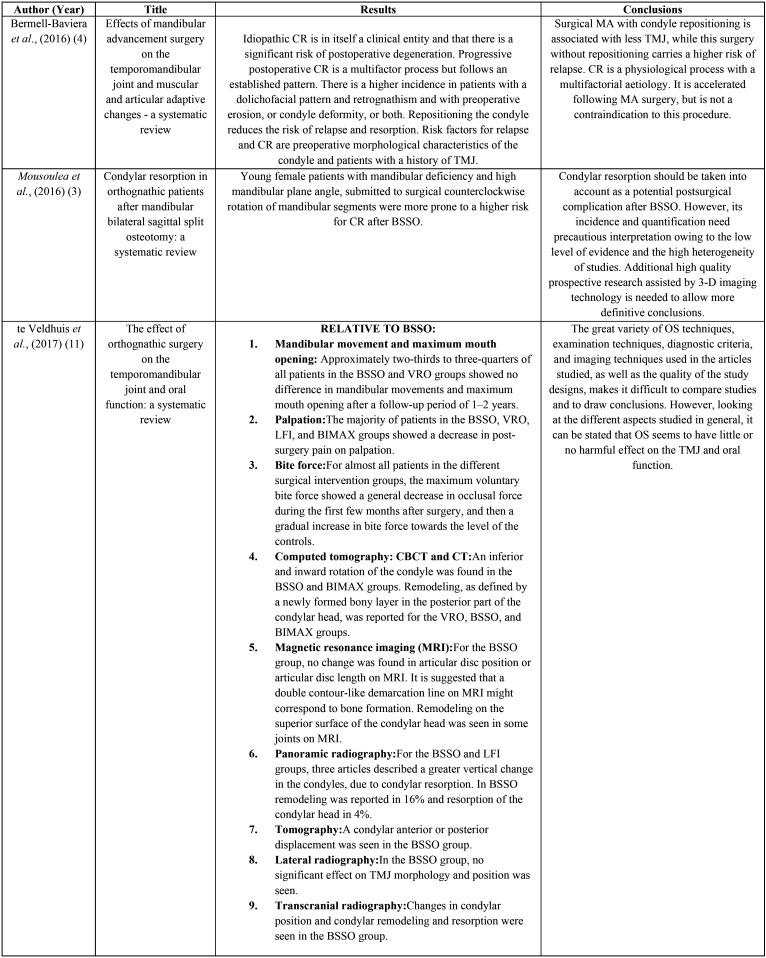
Results and Conclusions of the included studies ordered by date.

**Table 3 cont.**
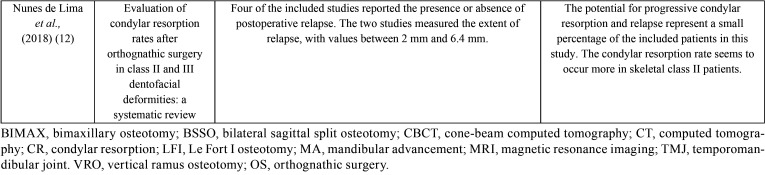
Results and Conclusions of the included studies ordered by date.

**Table 4:**
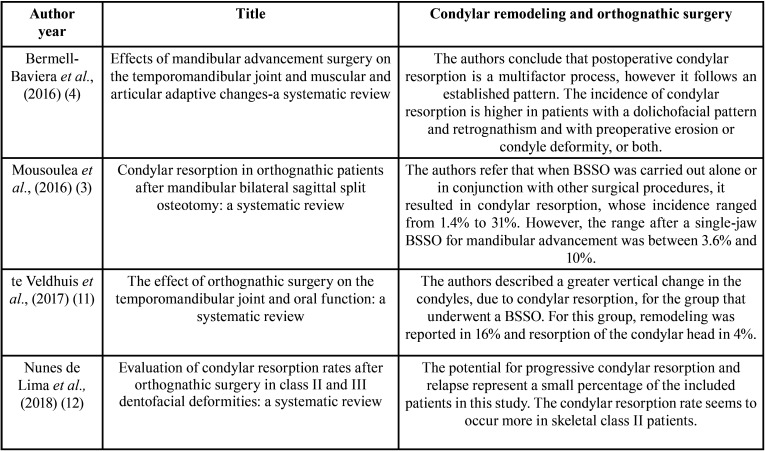
Correlation between condylar remodeling and orthognathic surgery.

**Figure 2:**
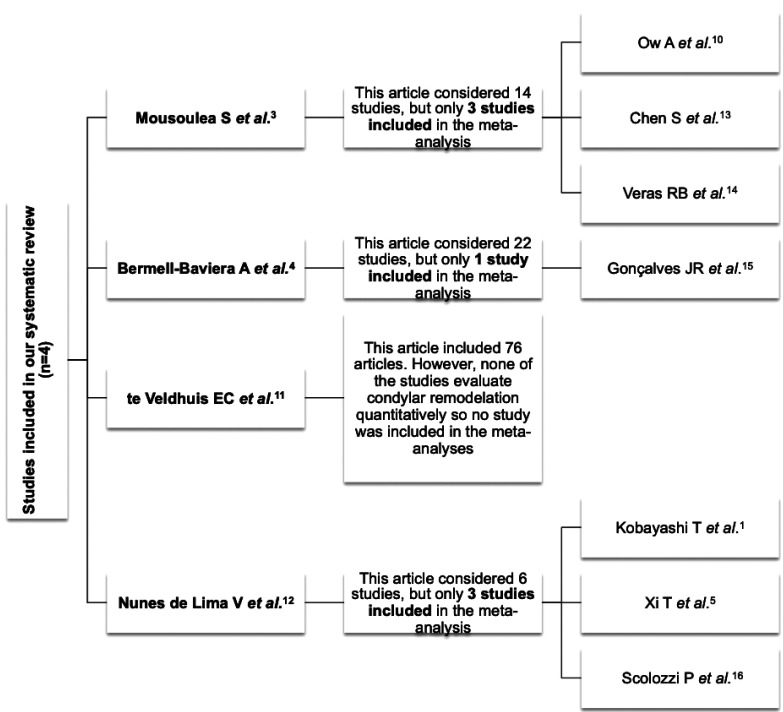
Flow diagram of included studies in the meta-analysis.

**Figure 3:**
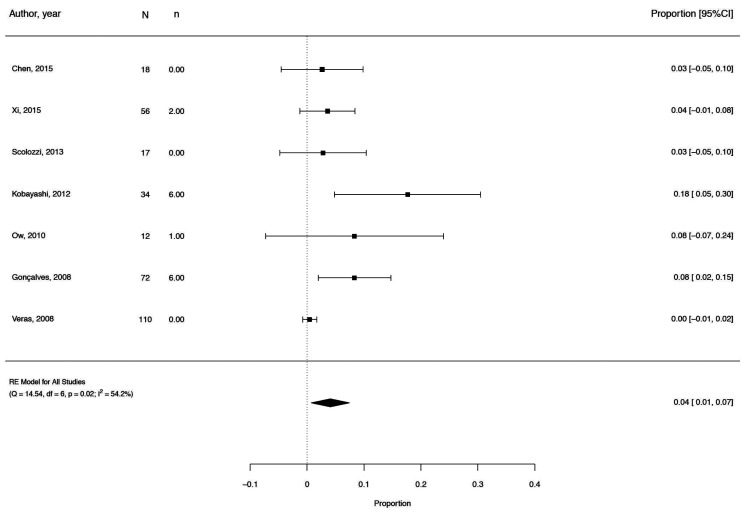
Forest plot of Meta-analysis.

The selected papers show some degree of heterogeneity (I2 = 55.85%; Q(3) = 6.788; *p* = 0.0791) and for that reason we chose a random effects model to estimate the global proportion value depicted in the forest plot (Figs. 2,3) (1,3-5,10-16).

## Discussion

The incidence of condylar form alteration is an outcome reported in previous reviews (2-5,11,17,18). However, the lack of meta-analysis concerning this subject was noted by the authors. Thus, we systematically evaluated and summarized evidence of data available with a statistical analysis from published SR.

The TMJ response to mandibular advancement ranged from adaptive, which included physiological bone remodeling, to irreversible complications (19).

The pathogenesis of condylar form alteration is not clearly identified, as this can be multifactorial. Therefore, it is not possible to recognize only one aetiological factor of TMJ changes. New unbalanced force vectors applied and absence of condylar adaptation may cause alterations on the condylar form. The internal rearrangement promotes disc displacement, with or without reduction, causing pressure on the articular surface during the rotation of the condyle, promoting the formation of TMD (12). One of the factors that may influence alteration of the condylar form is the magnitude of mandibular advancement and consequently the tension created from stretching the muscles as well as adjacent soft tissues. This tension may overcome the adaptive ability of the condyle creating compressive areas on the condylar head (1,3,20).

The small prevalence of alteration of the condylar form (OR = 0,04) may indicate that there is little influence on condylar alteration after mandibular advancement surgery (3,4,10,11).

The alteration of the condylar form is therefore more closely related to the surgical procedure in which there is counter clockwise rotation of the proximal mandibular segment, increasing the pressure on the less loaded surface of the anterior-superior condylar area (1). Deficit of blood supply appears to also have a role in condylar form alteration (3,21).

Our research highlights the lack of quantity and quality of articles that assess the alteration of the condylar form on skeletal class II patients that have been submitted to orthognathic surgery. Of the 37 identified articles 75,7% were not relevant, 16.2% were ineligible by applying the inclusion and exclusion criteria and also due to a negative quality evaluation. In conclusion, only 8.1% were considered for the final analysis.

All selected studies showed that the alteration of the condylar form could be a consequence of BSSO with mandibular advancement surgery. The systematic review published by Mousoulea *et al.* in 2017 concluded that young female patients with mandibular deficiency and high mandibular plane angle, submitted to surgical counterclockwise rotation of mandibular segments, were more prone to alteration of the condylar form after BSSO (3).

Veldhuis *et al.*, in 2017, identified condylar remodeling through cone-beam computed tomography (CBCT), computed tomography (TC), magnetic resonance imaging, panoramic radiography, lateral radiography and transcranial radiography. Panoramic radiography only identified condilar remodeling in patients that undergo BSSO. CBCT identified condilar remodeling in BSSO, vertical ramus osteotomy and bimaxillary osteotomy (11).

Another systematic review carried out by Bermell-Baviera *et al.*, in 2016, shows that when idiopathic condylar resorption is present there is a significant risk of postoperative degeneration. Progressive postoperative alteration of the condylar form is a multifactor process but seems to follow an established pattern. Additionally, there is a higher incidence in patients with a dolichofacial pattern and retrognathism and with preoperative erosion, or condyle deformity, or both. Furthermore, repositioning of the condyle reduces the risk of relapse and alteration of its form. Preoperative morphological characteristics of the condyle and patients with a history of TMD are risk factors for relapse and alteration of the condylar form (4).

Of the 3 studies included, Bermell-Baviera *et al.* in 2016, Mousoulea *et al.* in 2016 and te Veldhuis *et al*. in 2017, all reported the use of CBCT to identify the alteration of the condylar form. As the design of the included studies were systematic reviews, some articles identified by them evaluated condylar resorption with 2-dimensional imaging techniques. Mousoules *et al.* concluded that additional high quality prospective research assisted by 3D- imaging technology is needed to allow more definitive conclusions (3,4,10,11).

Even though 2-D imaging is commonly used, it cannot be considered the gold standard for evaluation of alteration of the condylar form. On the other hand, the magnification of 3-D imaging allows a superimposition and comparison of the condyles, enabling the correct assessment of condylar changes (resorption or remodeling) (3,5,22).

All selected publications it is agreed that in the treatment of skeletal class II, after BSSO with mandibular advancement, alteration of the condylar form can occur (21). However, this finding should be treated with caution, as there is a considerable heterogeneity of the studies regarding this matter. The heterogeneity of these studies might be explained by differences in the study designs. Even though we used four systematic reviews, that included cohort studies (retrospective and prospective) (3,4,11).

The types of surgical interventions included in the four articles are not homogeneous: Bermell-Baviera *et al.*, evaluate only studies with BSSO mandibular advancement surgery, whereas the other two studies, included articles where BSSO surgery was performed with other surgical procedures, such as Le Fort I (4).

A qualitative assessment was performed using AMSTAR 2 analysis, which provide methodological quality of included articles.

In this review we defined very strict inclusion and exclusion criteria, which may influence the results obtained. The heterogeneity found in the included studies is also a limitation.

To conduct this overview, the authors only include systematic reviews, excluding randomized and non-randomized controlled trials and cohort studies. Therefore, our review sample was low because of methological problems in the assessed papers. Most studies showed no randomization of their sample, as it is difficult to randomize a surgical procedure.

Another important limitation of the quality assessment was the lack of methods for quantifying the alteration of the condylar. Future study designs should randomize their samples from the beginning fostering their evidence, as is mentioned on the CONSORT (Consolidated Standards of Reporting Trials) guidelines. However, for ethical reasons, randomized clinical trial designs involving surgery are limited.

More studies on this topic are needed, particularly with more high-quality research.

## Conclusions

-Implications for clinical practice

Base on the meta-analysis, the alteration of the condylar form may be a consequence of BSSO with mandibular advancement surgery. Due to the aforementioned limitations of the methodology, these results should be taken carefully. Additional high quality prospective research assisted by 3D-imaging technology is needed to allow more definite conclusions.

-Implications for Research

These findings clearly demonstrate the necessity of further randomized controlled trials in order to evaluate not just the condylar resorption presence but also to quantify and differentiate the changes in the condyle (remodeling and resorption).
